# Assessing associations amongst body dissatisfaction, eating disorder symptoms and sociocultural influences in adolescents from rural Nicaragua

**DOI:** 10.1186/s40359-025-03728-3

**Published:** 2025-12-06

**Authors:** Fabienne E. Andres, Tracey Thornborrow, Wienis N. Bowie, Malcolm Gonzalez, Elizabeth H. Evans, Lynda G. Boothroyd

**Affiliations:** 1 Department of Psychology, Durham University, Durham, UK; 2 School of Psychology, University of Lincoln, Lincoln, UK; 3 Universidad de las Regiones Autónomas de la Costa Caribe Nicaragüense (URACCAN), Bluefields, Nicaragua

**Keywords:** Body image, Media internalization, Sociocultural influences, Eating disorder symptoms, Nicaragua, Ethnic identity

## Abstract

**Introduction:**

We conducted a cross-sectional study with adolescents in rural Nicaragua to assess the associations amongst body dissatisfaction, eating disorder symptoms and sociocultural influences (sociocultural pressures and media internalization), and assessed gender and ethnic group differences.

**Methods:**

A total of 122 adolescents aged 11–18 years (77 girls; *M*_age_ = 13.8, *SD* = 1.55) from small rural communities completed validated, culturally adapted questionnaires measuring perceived sociocultural pressures, media internalization, body dissatisfaction and eating disorder symptoms.

**Results:**

Stronger perceived sociocultural pressures and greater media internalization were associated with higher levels of body dissatisfaction and more eating disorder symptoms in both boys and girls. Perceived sociocultural pressure from family, friends and the media was the strongest predictor of body dissatisfaction in hierarchical regression models. Although general media internalization did not predict body dissatisfaction, athletic internalization emerged as a significant predictor for girls in exploratory analyses. Girls showed lower body dissatisfaction and lower media internalization compared to boys. Compared to participants of Afro-descended ethnicity, Indigenous participants showed higher body dissatisfaction, higher media internalization and higher perceived sociocultural pressure. There were no gender or ethnic differences in eating disorder symptoms.

**Conclusions:**

Our results suggest that perceived pressures from family, friends and the media are stronger predictors for body dissatisfaction than media internalization - for both boys and girls. Furthermore, differences between Indigenous and Afro-descended participants highlight the importance of taking cultural context and ethnicity into account when assessing body dissatisfaction and eating disorder symptoms in rural, Latin American populations.

**Supplementary Information:**

The online version contains supplementary material available at 10.1186/s40359-025-03728-3.

## Background

The current study investigated associations between sociocultural influences on body dissatisfaction and eating disorder symptoms in adolescents from the Caribbean coast of Nicaragua. Body dissatisfaction occurs when the discrepancy between an individual’s perceived and ideal body size and shape provokes negative thoughts and feelings [1]. Body dissatisfaction is prevalent in Latin America and around the world [2–5]. A study examining 2480 Brazilian adolescents found that none of them viewed their weight as ideal, with most expressing a desire to be thinner [6]. Body dissatisfaction is a key risk factor for developing eating disorders [7], which are considered a significant public health burden and are associated with high economic costs [8, 9]. Prevalence of eating disorders is increasing in Latin America, with the prevalence of Bulimia nervosa and Binge-eating disorder exceeding the US and Western Europe [10, 11]. Further understanding the factors that are associated with body dissatisfaction and eating disorder symptoms in Latin American adolescents, is therefore essential.

Adolescence is a period of intense and rapid physical, psychological, cognitive, emotional and social change and a period of weight gain, especially in girls [12]. This puts them at high risk for the development of body dissatisfaction and related psychopathology including eating disorders and depressive disorders [13–15]. Research indicates that during early adolescence, girls tend to report a decline in body satisfaction, with body dissatisfaction reaching its peak around age 16 [16, 17]. Conversely, boys generally experience an increase in body satisfaction during early adolescence, which then stabilizes after the age of 16 [12, 17]. This development is coupled with the increasing importance of sociocultural influences (including messages about appearance standards and perceived pressure from family, friends, and the media) on adolescents` developing self and body concepts. Sociocultural influences and especially media internalization are associated with body dissatisfaction in Western and Latin American populations [18–20].

Media consumption can significantly influence perceptions of appearance and preferences regarding body size. For instance, consumption of Western media (defined as TV, movies, magazines and music from the US, Canada or Western Europe) predicted preference for thinner bodies in both men and women, and body dissatisfaction in women across 26 countries [21]. The introduction of television in Fiji led to increased body concerns and eating disorders among Fijian adolescents [22]. Studies conducted on the Caribbean Coast of Nicaragua showed that TV consumption was associated with: women preferring both slimmer and curvier ideal body shapes for women [23, 24]; men preferring slimmer women with larger breasts [25]; and a higher likelihood of women dieting [26].

The sociocultural model of eating pathology proposes that internalization (e.g., the extent to which an individual ‘buys into’ a culturally prevalent appearance standard) of a thin or athletic body ideal creates this perceived discrepancy which can lead to body dissatisfaction, and potentially increase the risk for eating disorder symptoms via dietary restraint and low mood [27]. This model has been supported in Western samples of children and adolescents [28–30] and in urban adolescent and adult populations in Latin America in cross-sectional [5, 31] and longitudinal [32] studies. In rural Nicaraguan women, body shape concerns fully mediated the effect of thin-ideal internalization on eating disorder symptoms in cross-sectional data [33], suggesting that the sociocultural model may be useful in rural/low SES populations in Latin America.

Vulnerability to media pressure and internalization of media ideals can vary across ethnic, socioeconomic groups and gender within a population. However, studies from Latin America are scarce and most studies assessing the associations between body image and media influence in Latin America did not include ethnic identity or socioeconomic status, and were limited to recruiting urban samples [18]. Studies from rural Nicaragua showed that Mestizo (predominantly of Spanish European descent) women showed higher media internalization and preferred slimmer bodies than Creole (Afro-Caribbean descent) women, even though the latter watched more TV [23, 33]. In the same region, Indigenous Miskitu men showed higher drive for muscularity than Creole and Mestizo men [34]. Women from an Indigenous group living in rural Guatemala reported higher body satisfaction, compared to participants living in urban Colombia [35]. Two studies investigating the influence of media on appearance ideals and body dissatisfaction in rural Argentina found limited evidence for TV influence on body size ideals, and no associations between body dissatisfaction and media influence in adults [36, 37]. To our knowledge, only one study assessed the prevalence of eating disorder symptoms in Indigenous populations in Latin America. Indigenous men and women from Colombia reported even more eating disorder symptoms than the general population, with the risk being twice as high for Indigenous participants living in urban settings [38]. Regarding differences by SES, research found that higher SES is associated with a thinner body ideal among Colombian women [39] and adolescent girls in Brazil [40–42]. However, other studies have not found differences in body satisfaction by SES in Brazil [43].

Research suggests that men and boys typically exhibit lower body dissatisfaction and eating disorder symptoms compared to women and girls [44, 45]. Brazilian adolescent boys showed slightly lower correlations between media internalization scores and body dissatisfaction, compared to girls [4, 42]. However, gender did not moderate the relationship between body satisfaction and social media use in a recent meta-analysis [46]. Further, two longitudinal studies from Australia and Germany suggested that the associations between media (both athletic and thin) internalization, body dissatisfaction and eating disorder symptoms were similar among adolescent boys and girls [47, 48]. Media internalization was also a significant predictor of body dissatisfaction in Brazilian adolescent boys and girls one year later [32]. However, when men do experience body dissatisfaction, it tends to centre around muscularity rather than leanness [49]. For example, drive for muscularity was associated with lower body satisfaction and higher media internalization in men from Brazil and Mexico [50, 51] and with more internet use in adult men from Argentina [52]. Rural Nicaraguan men also reported feeling pressure to have a muscular body [34].

### The current study

In this study, we cross-sectionally assessed associations of the sociocultural model of eating disorders and body image between body dissatisfaction, eating disorder symptoms, and sociocultural influences, and considered gender and ethnic differences in adolescents. All data were gathered during the initial assessment phase of a pilot study of a school-based body image program in Nicaragua. All data were collected in four rural communities of the Pearl Lagoon Basin on the Caribbean Coast of Nicaragua. Out of these four communities, only one is accessible by road, whereas the three others are only accessible by water. Two of them were connected to the national electricity grid, the other two relied on part-time generators, providing between 10 and 16 h of electricity per day. Most of the region’s population in the communities relies on fishing, farming, and low-wage jobs for their livelihood, often receiving additional financial support from family members who have migrated abroad. Food insecurity is notably prevalent in this area [24]. The Caribbean Coast of Nicaragua experienced rapid increases in connectivity and infrastructure, which dramatically increased media consumption and access to social media in this region over the last ten years [23].

## Materials & methods

### Participants

Our sample consisted of 122 adolescents (77 girls, *M*_age_ = 13.8, *SD* = 1.55, range = 11–18). A total of 48% of participants identified as belonging to Indigenous ethnic groups (Ulwa and Miskitu), 45% as belonging to Afro-descended ethnic groups (Creole and Garifuna) and 7% as Mestizo ethnicity (mostly Spanish European descent). In three of the communities, we collected data from nearly all adolescents attending 7th and 8th grade. Participants reported the mean time spent on social media per week was 22.5 h (*SD* = 26.5) and 6.4 h (*SD* = 9.9) watching TV per week.

### Procedure

Data were collected during the baseline phase of a pilot study testing a school-based body image programme between February and May 2022. This was a collaborative project between the Universidad de las Regiones Autónomas de la Costa Caribe Nicaragüense (URACCAN) and Durham University. All data were collected in accordance with the Declaration of Helsinki and ethical approval was received from the Department of Psychology Ethics Sub-committee at Durham University (reference *PSYCH-2020-08−20T11:47:35-dfls13).* The study also received approval from the Nicaraguan Ministry of Education’s regional office (Ministerio de Educación, MINED) and community leaders. In line with local protocol, head teachers gave consent for their students to participate. Parents were informed about the study via flyers and communal meetings and could opt-out their children from participating if desired. Students gave written assent before completing the paper questionnaire packs in the classroom.To facilitate comprehension among adolescents with weaker reading skills, WB & MG read each item out aloud to ensure comprehension. However, students also had the option to read and respond to the questions at their own pace.

### Measures

All questionnaire items were provided in both Spanish and English (Creole) to further improve student understanding (the official national language is Spanish, however in this area the first language is typically Creole English or Miskitu). Some questionnaires were adapted based on previous data collected in this region [33] and further input from WB to reflect local terminology to improve understanding. All adaptations made are listed in supplementary material S1.

#### Body dissatisfaction

Participants completed the weight and appearance subscales of the Body Esteem Scale for Adults and Adolescents (BESAA; 53). The Latin American Spanish version has been validated in Colombian and Nicaraguan adults and showed excellent reliability in Nicaraguan adults (Cronbach’s α = 0.93) [53]. The BESAA measures the degree of satisfaction/dissatisfaction with general appearance and weight. These two subscales constitute of 18 positively and negatively worded statements about weight and appearance (e.g., “I like what I look like in pictures”). Respondents answer using a 5-point scale from 0 (never) to 4 (always). A higher score indicates lower body dissatisfaction. Reliability of the BESAA in this sample was 0.74 (Cronbach’s α).

#### Media internalization

To measure media internalization, adapted versions of the subscales ‘general’ and ‘athletic’ internalization of the Sociocultural Attitudes Toward Appearance Questionnaire-3 (SATAQ-3; 55) were used. The Spanish version has been validated in Spanish adolescents and showed good reliability (general internalization Cronbach’s α = 0.84; athletic internalization Cronbach’s α = 0.78) [54]. Responses are given on a Likert scale from 1 (strongly disagree) to 5 (strongly agree) (e.g., general internalization: “I would like my body to look like the people who are in movies”, athletic: “I wish I looked as athletic as sports stars”). Higher total scores indicate greater media internalization. Based on earlier work, negatively worded items were removed to improve understanding [33, 34]. Additionally, as there are no magazines available in this region [23], references to ‘magazines’ in items were replaced with ‘social media’ (e.g., “I would like my body to look like people on social media”). Factor structure of this modified version was confirmed using Exploratory Structural Equation Model (ESEM), which yielded good fit indices and confirmed the suitability of the scale. ESEM fit indices are available in supplementary material S2. Cronbach’s alpha confirmed acceptable reliability for both general (Cronbach’s α =.76) and athletic internalization (Cronbach’s α =.75).

#### Perceived sociocultural pressures

The Perceived Sociocultural Pressure Scale (PSPS; 57) was used to assess pressure from media, family and friends to change body shape, weight and muscularity (e.g., “I’ve felt pressure to change my appearance from family/friends/media”) on a 5-point Likert scale from 1 (none) to 5 (a lot). Higher scores indicate higher perceived pressure. The scale has been translated and used in previous studies with Nicaraguan men [34] and has been pilot-tested with adolescents in this region (unpublished data). Cronbach’s alpha confirmed excellent reliability (Cronbach’s α = 0.91).

#### Eating disorder symptoms

An adapted version of the Eating Attitudes Test (EAT; 58) was used to assess eating disorder symptoms and attitudes. The Spanish version has been validated in Colombian women and found excellent reliability (Cronbach’s α = 0.92). Based on previous research [33, 55] and feedback from collaborators, we removed items unsuitable for the local context, resulting in the retention of 15 items (see supplementary material S1 for detailed explanations of the changes made). Additionally, the response options were reduced to a 5-point scale from 1 (never) to 5 (always) to improve participant understanding, as has been done in Colombia [56]. For analysis, continuous responses were used. This version showed acceptable reliability (Cronbach’s α = 0.75).

### Data analysis

Data were analysed using RStudio 4.0.3 [57]. The dataset supporting the conclusions of this article is available in the osf repository, (https://osf.io/enfa4). Firstly, we computed Pearson’s correlations to assess associations between all main variables. Secondly, we computed ANOVAs to assess differences by gender and ethnicity (between Afro-descent and Indigenous participants) in body dissatisfaction (BESAA), eating disorder symptoms (EAT), general internalization (SATAQ internalization-general), athletic internalization (SATAQ internalization-athletic), and perceived sociocultural pressures from family, peers and the media (PSPS). Finally, we ran a series of hierarchical multiple regression models to evaluate the individual contributions of our variables of interest. We assessed the impact of general internalization (SATAQ subscale) and perceived sociocultural pressures (PSPS) on body dissatisfaction (BESAA). Similarly, we assessed the impact of SATAQ general internalization, perceived sociocultural pressures (PSPS) and body dissatisfaction (BESAA) on eating disorder symptoms (EAT) by entering them subsequently in a hierarchical regression model. These analyses were run separately for boys and girls.

## Results

Zero order correlations (Pearson’s *r*) showed that among girls, body dissatisfaction scores were significantly and negatively correlated with athletic internalization, sociocultural pressure and eating disorder symptoms. Eating disorder symptom scores were significantly and positively correlated with general and athletic internalization, as well as sociocultural pressure. For boys, there were significant negative associations between body dissatisfaction, general and athletic internalization and sociocultural pressure, but not with eating disorder symptoms. Eating disorder symptoms were positively associated with athletic internalization and sociocultural pressure, but not with general internalization (see Table 1 for correlations, presented below the diagonal for girls and above the diagonal for boys).

**Table 1:** Zero-order correlations (Pearson’s r) between main variables separately for boys (above diagonal) and girls (below)

		1.	2.	3.	4.	5.
1.	BESAA		− 0.30*	− 0.33*	− 0.42**	− 0.27
2.	SATAQ - general	− 0.10		0.63**	0.40**	0.24
3.	SATAQ - athletic	− 0.33**	0.68**		0.32*	0.30*
4.	PSPS	− 0.37**	0.27*	0.32**		0.50**
5.	EAT	− 0.34**	0.28*	0.27*	0.64**	

* *p* <.05, ** *p* <.01. *BESAA* Body Esteem Scale for Adolescents and Adults, *SATAQ* Sociocultural Attitudes Towards Appearance Questionnaire general and athletic internalization subscales, *PSPS* Perceived Sociocultural Pressures Scale, *EAT* Eating Attitudes Test

**Fig. 1:**
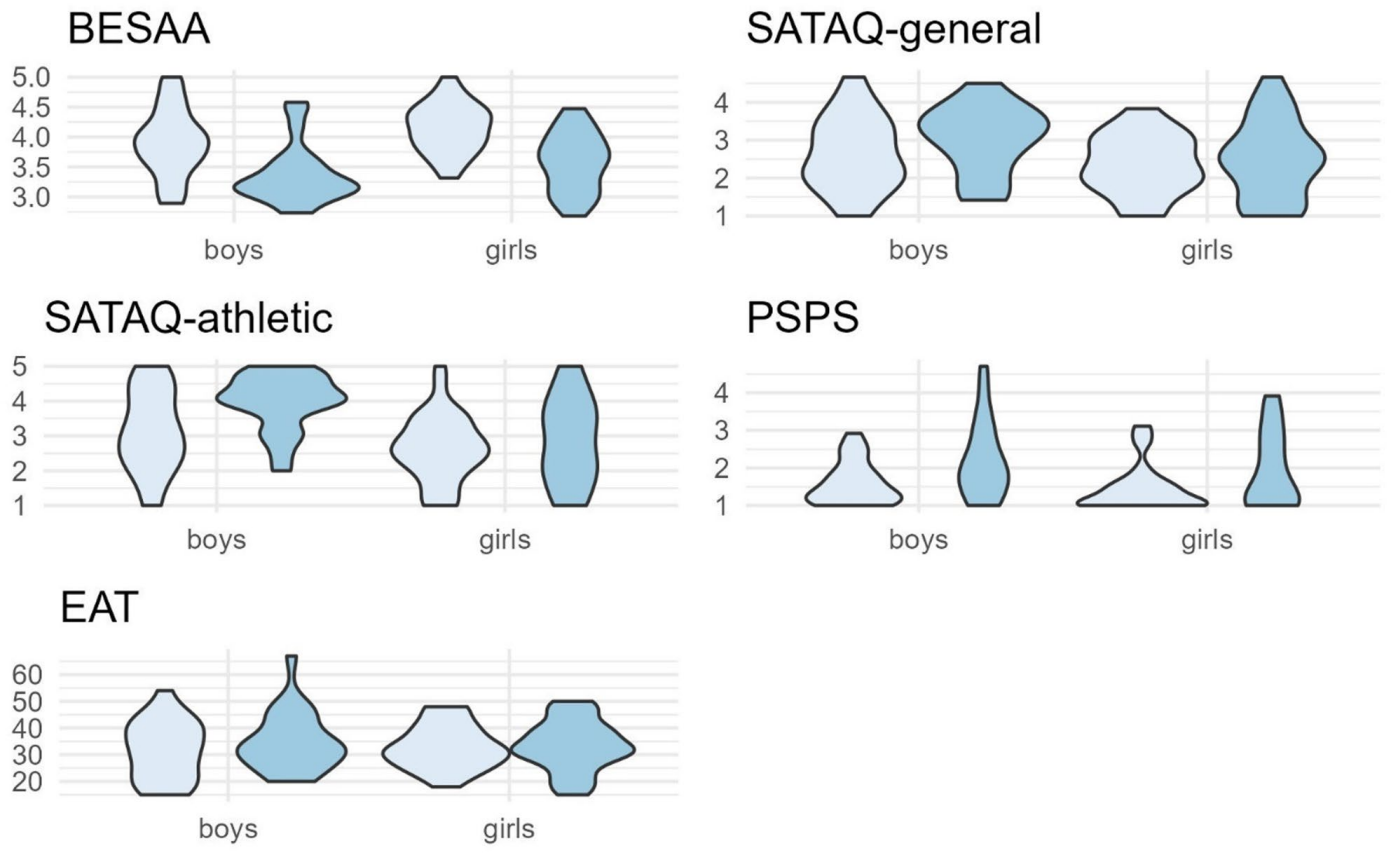
Distribution of mean scores in boys and girls by ethnicity (*N* = 114); light blue (left) represents Afro-descent participants, darker blue (right) represents Indigenous participants. BESAA = Body Esteem Scale for Adolescents and Adults, SATAQ = Sociocultural Attitudes Towards Appearance Questionnaire, general and athletic internalization subscales, PSPS = Perceived Sociocultural Pressures Scale, EAT = Eating Attitudes Test

ANOVA results showed that girls reported significantly higher body esteem than boys, indicating greater body dissatisfaction among boys. Afro descent participants reported significantly higher body esteem compared to Indigenous participants. Boys had significantly higher general and athletic internalization scores compared to girls. There were no gender differences for perceived sociocultural pressures. Indigenous participants showed significantly higher athletic internalization and higher sociocultural pressure from family, friends and media, compared to Afro-descent participants. There were no differences in eating disorder symptoms across gender or ethnicity. See Table 2; Fig. 1 for all results.

**Table 2:** Analyses of variance (ANOVAs) for differences by gender and ethnicity (Afro-descended vs. Indigenous) of body dissatisfaction, media internalization, perceived sociocultural pressure and eating disorder symptoms

	Gender	Ethnicity	Gender*Ethnicity
BESAA	F(1, 110) = 5.27*	F(1, 110) = 37.44***	F(1, 110) = 0.05
SATAQ gen. int.	F(1, 108) = 5.09*	F(1, 108) = 1.79	F(1, 108) = 0.44
SATAQ athl. Int.	F(1, 108) = 13.94***	F(1, 108) = 5.22*	F(1, 108) = 2.154
PSPS	F(1, 110) = 1.25	F(1, 110) = 12.01*	F(1, 110) = 0.09
EAT	F(1,110) = 2.36	F(1,110) = 0.008	F(1,110) = 0.027

* *p* <.05, ** *p* <.01, *** *p* <.001. *BESAA* Body Esteem Scale for Adolescents and Adults, *SATAQ* Sociocultural Attitudes Towards Appearance Questionnaire general and athletic internalization subscales, *PSPS* Perceived Sociocultural Pressures Scale, *EAT* Eating Attitudes Test

Hierarchical regression models with body dissatisfaction (BESAA) as outcome variable showed that higher media internalization and higher sociocultural pressure significantly predicted higher body dissatisfaction in boys. In girls, general internalization was not a significant predictor in any of the models. See Table 3 for results.

**Table 3:** Hierarchical regression analysis with body dissatisfaction (BESAA) as outcome, separate analysis for boys and girls

	B	SE B	Beta	t	F	df	R2
Girls							
SATAQ gen. int.	−0.07	0.08	−0.10	0.12	0.72	1, 71	0.010
SATAQ gen. int.	0.00	0.08	0.00	0.12	5.77***	2, 70	0.141
PSPS	−0.27**	0.08	−0.38	0.12			
Boys							
SATAQ gen. int.	−0.19*	0.09	−0.29	0.15	3.90*	1, 41	0.087
SATAQ gen. int.	−0.11	0.10	−0.18	0.16	3.64**	2, 40	0.154
PSPS	−0.21*	0.12	−0.28	0.16			

* *p* <.05, ** *p* <.01, *** *p* <.001. *BESAA* Body Esteem Scale for Adolescents and Adults, *SATAQ* Sociocultural Attitudes Towards Appearance Questionnaire, general internalization subscale, *PSPS* Perceived Sociocultural Pressures Scale

Regression models testing predictors of eating disorder symptoms (EAT) showed that only sociocultural pressure significantly predicted EAT scores; general internalization and body dissatisfaction scores did not significantly contribute to the full models in boys or girls. See Table 4 for results.

**Table 4:** Hierarchical regression analysis with eating disorder symptoms (EAT) as outcome, separate analysis for boys and girls

	B	SE B	Beta	t	F	df	R2
Girls							
SATAQ gen. int.	2.55*	1.16	0.25	0.11	4.85**	1, 71	0.064
SATAQ gen. int.	0.91	0.99	0.09	0.10	21.75***	2, 70	0.383
PSPS	6.57***	1.09	0.59	0.10			
SATAQ gen. int.	0.91	0.98	0.09	0.10	15.56	3. 69	0.403
PSPS	5.92***	1.16	0.53	0.10			
BESAA	−2.38	1.55	−0.15	0.10			
Boys							
SATAQ gen. int.	2.87	1.79	0.24	0.15	2.57	1, 41	0.059
SATAQ gen. int.	0.23	1.71	0.02	0.14	8.72***	2, 40	0.304
PSPS	7.61***	2.03	0.54	0.14			
SATAQ gen. int.	0.05	1.75	0.00	0.15	5.85***	3, 39	0.310
PSPS	7.26**	2.13	−0.09	0.14			
BESAA	−1.68	2.72	−0.09	0.14			

* *p* <.05, ** *p* <.01, *** *p* <.001. *EAT* Eating Attitudes Scale, *SATAQ* Sociocultural Attitudes Towards Appearance Questionnaire general internalization subscale, *PSPS* Perceived Sociocultural Pressures Scale, *BESAA* Body Esteem Scale for Adolescents and Adults

Since we found higher levels of athletic internalization relative to general internalization in both boys and girls, we ran additional exploratory hierarchical regression models with the athletic internalization subscale instead of the general internalization subscale. Results are presented in supplementary materials S3. The only way in which these models differed from those presented here was that for girls, the athletic internalization subscale significantly predicted body dissatisfaction in the full model.

## Discussion

This cross-sectional study assessed the relationships amongst perceived sociocultural pressures, media internalization, body dissatisfaction, and eating disorder symptoms in an ethnically diverse sample of adolescents in rural Nicaragua. To our knowledge, the current study is the first to investigate the associations between body dissatisfaction, eating disorder symptoms and sociocultural influences in Latin American adolescents from rural communities. We found significantly higher media internalization (general and athletic ideals) and body dissatisfaction in boys compared to girls. This pattern differs from results in other Latin American (and most Western) samples where girls generally show higher body dissatisfaction and higher media internalization than boys (Brazil: 32, Argentina: 62, Mexico: 63). Our results align with findings from adults in this region, where men exhibited higher general internalization of appearance ideal scores than women [55]. One explanation for lower body dissatisfaction and general media internalization scores in Nicaraguan girls could be a strong emphasis on cultural practices and ethnic identity, which include a strong feeling of belonging to their (minority) ethnic group and loving the body they have [58], which could be a potentially protective factor for Nicaraguan girls.

When considering ethnic differences, Afro-descended adolescents demonstrated lower perceived sociocultural pressure, lower internalization of media ideals and lower body dissatisfaction than Indigenous adolescents. This is consistent with previous data from adult Indigenous (Miskitu) men, who showed higher body dissatisfaction and higher drive for muscularity compared to their Afro-descended (Creole) compatriots [34]. Several studies from the US have shown that Black men and women reported lower body dissatisfaction and eating disorder symptoms compared to White or Hispanic individuals [59–61]. It has been suggested that individuals belonging to an Afro-descended ethnic group might feel less pressure to conform to white-dominated appearance standards portrayed in the media, and are more accepting of a wider range of appearance standards [62, 63]. We did not find significant differences in eating disorder symptoms for Indigenous and Afro-descended participants. Indigenous participants have been shown to be at risk for the development of body dissatisfaction and eating disorder symptoms. For instance, the majority of Mexican Indigenous women reported being dissatisfied with their weight, regardless of their BMI [64] and Indigenous men and women from Colombia reported higher eating disorder symptoms than the general population of Colombia [38]. However, more research is needed among Indigenous ethnic groups to assess their vulnerability to media pressure and internalization, and potential pathways to body dissatisfaction and eating disorder symptoms in Latin American adolescents.

Deviating from the associations of the sociocultural model of body image and eating disorders, we found that appearance pressure from family, friends and the media was the strongest predictor of body dissatisfaction in our regression models in both Nicaraguan boys and girls, and not media internalization. Additionally, sociocultural pressure was also the strongest predictor for eating disorder symptoms, whereas internalization and body dissatisfaction were not significant. One explanation might be the high frequency of appearance comments people tend to make (personal observation made by FA and TT), which might have a greater effect on adolescents than internalized media ideals. Future qualitative research could explore whether frequent appearance-related comments in everyday interactions contribute to adolescent body dissatisfaction. Unexpectedly, athletic rather than general internalization predicted body dissatisfaction among girls in the regression models. This may indicate that girls in this context value or engage more with media imagery of athletes and sports stars, where focus lies more on muscularity, compared to ‘traditional Western-like’ appearance ideals that mostly focus on thinness. Our results contrast with findings from adult Creole and Mestizo women in this region, where general internalization of appearance ideals did predict body dissatisfaction and eating disorder symptoms [33]. However, that study measured TV consumption as, at that time, local people did not use social media, which may explain differences found in sociocultural pressures and the degree of media internalization. Furthermore, some studies suggest that the muscular ideal may be more relevant for Black women than the thin ideal [65].

### Study strengths, limitations and future considerations

This study has several notable strengths. We recruited adolescents from an underrepresented, hard-to-reach population and recruited the majority of adolescents in three of the four communities on the Caribbean coast of Nicaragua. Based on pilot work, we culturally adapted questionnaires to ensure items were suitable for the specific linguistic and cultural context. Factor analysis showed acceptable fit indices for the original factor structures of the SATAQ. Culturally adapting measures for specific populations may limit comparisons with other samples or the generalizability of these findings to other populations. Further psychometric validation of the adapted measures is needed to ensure construct equivalency with the original versions. Our study was cross-sectional; to establish direction of causality, longitudinal research is needed. Although we achieved near-complete sampling in three communities, the findings may not generalize to all rural Nicaraguan adolescents or other ethnic groups.

Future studies should recruit larger sample sizes to evaluate the full sociocultural model and/or tripartite model using path analysis (as evaluated in Brazilian women; 67). Additionally, other studies sampling in this region have highlighted the importance of body shape, and not solely body size [25]. Therefore, it would be useful to additionally assess internalization of curvy ideals, employing for example the Curvy Ideal Internalization Scale (CII; 72).

## Conclusion

We investigated the associations between sociocultural pressures, media internalization, body dissatisfaction, and eating disorder symptoms in an ethnically diverse adolescent population from rural Nicaragua. Findings showed that participants who felt more sociocultural pressure and had greater media internalization showed increased body dissatisfaction and more eating disorder symptoms. Girls showed lower body dissatisfaction and media internalization than boys, while Indigenous participants reported higher levels of these variables—and greater sociocultural pressure—compared to their Afro-descended peers, with no observed differences in eating disorder symptoms across gender or ethnicity. Our results suggest that perceived appearance pressure may be a particularly salient correlate of body dissatisfaction and disordered eating in this context, possibly more so than internalized media ideals.

## Supplementary Information

Supplementary Material 1

Supplementary Material 2

Supplementary Material 3

## Data Availability

Anonymised data and analytic R code are available at: https://osf.io/enfa4.
