# Supplementary material for: Assessing associations amongst body dissatisfaction, eating disorder symptoms and sociocultural influences in adolescents from rural Nicaragua
Source: BMC Psychol. 2025 Dec 6;14:32. doi: 10.1186/s40359-025-03728-3 (PMC12797590; doi:10.1186/s40359-025-03728-3)
Supplement: Supplementary file 1 — Supplementary Material 1 [file 40359_2025_3728_MOESM1_ESM.docx]

# **S1 – translated and adapted questionnaires**

## Body esteem scale for adolescents and adults (BESAA; Mendelson, Mendelson & White, 2001)

| **Original version** | **Adapted Creole version** | **Comments** |
| --- | --- | --- |
| I like what I look like in pictures. | I like what I look like in photos | Pictures mean videos in Creole |
| I am proud of my body. | I am proud of my body |  |
| I am preoccupied with trying to change my body  weight. | I spend a lot of time trying to change my body weight | The term preoccupied has not been understood my locals and was therefore changed to “spending a lot of time” |
| I like what I see when I look in the mirror. | I like what I see when I look in the looking glass | Mirror is called looking glass |
| There are lots of things I’d change about my looks  if I could. | There are lots of things I’d change about my looks if I could |  |
| I am satisfied with my weight. | I am satisfied with my weight |  |
| I wish I looked better. | I wish I looked better |  |
| I really like what I weigh. | I really like what I weigh |  |
| I wish I looked like someone else. | I wish I looked like someone else |  |
| My looks upset me. | My looks upset me |  |
| I’m as nice looking as most people. | I’m as nice looking as most people |  |
| I’m pretty happy about the way I look. | I’m happy enough about the way I look | The term pretty happy was not understood |
| I feel I weigh the right amount for my height. | I feel I weigh the right amount for my height |  |
| I feel ashamed of how I look. | I feel ashamed of how I look |  |
| Weighing myself depresses me. | Weighing myself depresses me |  |
| My weight makes me unhappy. | My weight makes me unhappy |  |
| I worry about the way I look. | I worry about the way I look |  |
| I think I have a good body | I think I have a good body |  |
| I’m looking as nice as I’d like to. | I’m looking as nice as I’d like to |  |

## Eating Attitudes Test-26 (EAT-26; Constaín et al., 2014)

Out of the 26 items, 9 items were exclude because they were deemed not suitable for the local context, either because the concepts would need too much explaining because definitions are not clear (e.g. self-control, calories) or they do not apply in local eating traditions (e.g. cut food in small pieces).

| **Original** | **Creole English** | **Comments** |
| --- | --- | --- |
| 1 Am terrified about being overweight | 1 I am terrified about being overweight. |  |
| 2 Avoid eating when I am hungry. | 2 I avoid eating when I am hungry. |  |
| 3 Find myself preoccupied with food. | 3 I spend a lot of time thinking about food. | The term preoccupied was not understood, so we translated it as spending a lot of time |
| 4 Have gone on eating binges where I felt that I may not be able to stop. | 4 I have eaten a lot and felt like I couldn’t stop | It was not clear what eating binges were |
| 5 Cut my food into small pieces. |  | People mostly eat with a big spoon and do not cut their food |
| 6 Aware of the calorie content of foods that I eat. |  | A lot of people do not know what calories are |
| 7 Particularly avoid food with a high carbohydrates content (i.e. bread, rice, potatoes, etc.) |  | Local dishes always contain rice and locals got really confused about this question |
| 8 Feel that others would prefer if I ate more | 5 I feel that others would prefer it if I ate more. |  |
| 9 Vomit after I have eaten |  | When asked this, focus is on food poisoning and question was not suitable |
| 10 Feel extremely guilty after eating | 6 I feel very guilty after eating. | Extremely is not a term used in local language |
| 11 Am preoccupied with a desire to be thinner | 7 I spend a lot of time thinking about wanting to be more meagre. | Again, preoccupied is not understood. They use the word meagre instead of thinner |
| 12 Think about burning up calories when I exercise. | 8 I think about burning up energy (calories) when I exercise. | In order to understand the question, energy was added to explain what calories are |
| 13 Other people think that I am too thin |  | Being thin is not seen as pathological in the same sense as here and was confusing for participants |
| 14 Am preoccupied with the thought of having fat on my body. | 9 I am worried about the idea of having fat on my body. | Preoccupied is not understood; also changed thought to idea, because this was confusing to participants |
| 15 Take longer than others to eat my meals |  | People do not all sit at the same table to eat their meals, which makes comparisons with others less accurate |
| 16 Avoid foods with sugar in them | 10 I avoid foods with sugar in them. |  |
| 17 Eat diet foods. |  | Diet foods do not exist in the communities |
| 18 Feel that food controls my life. | 11 I feel that food controls my life. |  |
| 19 Display self-control around food. |  | Self-control as a concept is not known and would need to be defined first |
| 20 Feel that others pressure me to eat | 12 I feel that others pressure me to eat. |  |
| 21 Give too much time and thought to food. | 13 I give too much time and thought to food. |  |
| 22 Feel uncomfortable eating sweets. |  |  |
| 23 Engage in dieting behaviour. |  | They do not know what dieting behaviour means |
| 24 Like my stomach to be empty | 14 I like my stomach to be empty. |  |
| 25 Have the impulse to vomit after meals | 15 I have the impulse to vomit after meals. |  |
| 26 Enjoy trying new rich foods. |  | Food choices are limited in the communities and question does not make a lot of sense |

Sociocultural Attitudes Toward Appearance Quesionnaire-3 (SATAQ-3;Calogero et al., 2004; Sanchez-Carracedo et al., 2012)

All negatively worded items have been excluded because they were confusing to participants. Additionally, there are no magazines in this region. All items that would normally refer to magazines have been replaced with social media.

|  |
| --- |
| Los programas de TV y peliculas son fuentes importantes de información sobre la moda y el “ser atractiva.”  *TV and movies are important sources of information about fashion and “being attractive.”* |
| No me importa si mi cuerpo se parece al cuerpo de la gente que aparece en TV/ internet *I do not care if my body looks like the body of people who are on TV/the internet.* |
| Comparo mi cuerpo con los cuerpos de la gente que aparece en televisión. *I compare my body to the bodies of people who are on TV.* |
| Las redes sociales son fuentes importantes de información sobre la moda y el “ser atractiva.” *Social media are an important source of information on fashion and “being attractive.”* |
| Me gustaría que mi cuerpo se pareciera al cuerpo de las modelos que aparecen en las redes sociales. *I would like my body to look like people in social media.* |
| Comparo mi apariencia con la apariencia de las estrellas de televisión o del cine. *I compare my appearance to the appearance of TV and movie stars.* |
| Me gustaría que mi cuerpo se pareciera al cuerpo de la gente que sale en las películas. *I would like my body to look like the people who are in movies.* |
| Desearía parecerme a las modelos de los vídeos musicales.  *I wish I looked like the models in music videos.* |
| Comparo mi apariencia con la apariencia de la gente en las redes sociales. *I compare my appearance to the appearance of people in social media.* |
| Comparo mi cuerpo con el de la gente que está en “buena forma.” *I compare my body to that of people in “good shape."* |
| Deseo parecer tan atlética como las estrellas del deporte. *I wish I looked as athletic as sports stars.* |
| Comparo mi cuerpo con el cuerpo de la gente que es atlética. *I compare my body to that of people who are athletic.* |
| La gente famosa es una fuente importante de información sobre la moda y el “ser atractiva.” *Famous people are an important source of information about fashion and “being attractive.”* |
| Intento parecerme a las atletas deportivas. *I try to look like sports athletes.* |

## Perceived sociocultural pressure scale (PSPS; Stice, Ziemba, et al., 1996)

Own translation

Answer possibilities pressure questions: None, a little, some, quite a lot, a lot

Answer possibilities upset: Not upset, not very upset, a little upset, upset, very upset

| He sentido la presión de PERDER PESO de ...  *I’ve felt pressure to LOSE WEIGHT from…* | | | | | |
| --- | --- | --- | --- | --- | --- |
|  | Ninguna  *None* | Un poco  *A little* | Algo  *Some* | Bastante  *Quite a lot* | Mucho  *A lot* |
| 1…mis amigos/amigas  ….*my friends* |  |  |  |  |  |
| 2…mi familia … *my family* |  |  |  |  |  |
| 3…. los medios (p. Ej., Televisión, internet) *…the media (e.g., TV, internet)* |  |  |  |  |  |
| 4. ¿Qué tan molesto está por esta presión para perder peso?  *How upset are you by this pressure to lose weight?* |  |  |  |  |  |
| He sentido la presión de tener MÚSCULOS MÁS GRANDES de ...  *I’ve felt pressure to have BIGGER MUSCLES from…* | | | | | |
|  | Ninguna  *None* | Un poco  *A little* | Algo  *Some* | Bastante  *Quite a lot* | Mucho  *A lot* |
| 5…mis amigos/amigas  ….*my friends* |  |  |  |  |  |
| 6…mi familia … *my family* |  |  |  |  |  |
| 7…. los medios (p. Ej., Televisión, Internet) *…the media (e.g., TV, internet)* |  |  |  |  |  |
| 8 ¿Qué tan molesto estás por esta presión de tener músculos más grandes?  *How upset are you by this pressure to have bigger muscles?* |  |  |  |  |  |
|  | | | | | |
| He sentido la presión de CAMBIAR LA FORMA DE MI CUERPO...  I’ve felt pressure to CHANGE MY BODY SHAPE from… | | | | | |
|  | Ninguna  *None* | Un poco  *A little* | Algo  *Some* | Bastante  *Quite a lot* | Mucho  *A lot* |
| 9…mis amigos/amigas  ….*my friends* |  |  |  |  |  |
| 10…mi familia … *my family* |  |  |  |  |  |
| 11…. los medios (p. Ej., Televisión, Internet) *…the media (e.g., TV, internet)* |  |  |  |  |  |
| 12. ¿Qué tan molesto está por esta presión para cambiar la forma o la constitución de su cuerpo?  *How upset are you by this pressure to change your body shape or build?* |  |  |  |  |  |
| He sentido la presión de cambiar MI APARIENCIA de ... *I’ve felt pressure to change MY APPEARANCE from…* | | | | | |
|  | **Ninguna**  ***None*** | **Un poco**  ***A little*** | **Algo**  ***Some*** | **Bastante**  ***Quite a lot*** | **Mucho**  ***A lot*** |
| 13…mis amigos/amigas  ….*my friends* |  |  |  |  |  |
| 14…mi familia … *my family* |  |  |  |  |  |
| 15…. los medios (p. Ej., Televisión, Internet) *…the media (e.g., TV, internet)* |  |  |  |  |  |
| 16. ¿Qué tan molesto está por esta presión para cambiar su apariencia?  *How upset are you by this pressure to change your appearance?* |  |  |  |  |  |

# **References**

Calogero, R. M., Davis, W. N., & Thompson, J. K. (2004). The Sociocultural Attitudes Toward Appearance Questionnaire (SATAQ-3): Reliability and normative comparisons of eating disordered patients. *Body Image, 1*(2), 193-198. <https://doi.org/10.1016/j.bodyim.2004.01.004>

Constaín, G. A., Ramírez, C. R., de los Ángeles Rodríguez-Gázquez, M., Gómez, M. Á., Múnera, C. M., & Acosta, C. A. (2014). Validez y utilidad diagnóstica de la escala EAT-26 para la evaluación del riesgo de trastornos de la conducta alimentaria en población femenina de Medellín, Colombia. *Atención Primaria, 46*(6), 283-289. <https://doi.org/10.1016/j.aprim.2013.11.009>

Góngora, V. C., Licea, V. C., Chams, M. R. M., & Thornborrow, T. (2020). Assessing the measurement invariance of a Latin-American Spanish translation of the Body Appreciation Scale-2 in Mexican, Argentinean, and Colombian adolescents. *Body Image, 32*, 180-189. <https://doi.org/doi.org/10.1016/j.bodyim.2020.01.004>

Sanchez-Carracedo, D., Barrada, J. R., Lopez-Guimera, G., Fauquet, J., Almenara, C. A., & Trepat, E. (2012, Jan). Analysis of the factor structure of the Sociocultural Attitudes Towards Appearance Questionnaire (SATAQ-3) in Spanish secondary-school students through exploratory structural equation modeling. *Body Image, 9*(1), 163-171. <https://doi.org/10.1016/j.bodyim.2011.10.002>

Stice, E., Nemeroff, C., & Shaw, H. E. (1996). Test of the dual pathway model of bulimia nervosa: Evidence for dietary restraint and affect regulation mechanisms. *Journal of Social and Clinical Psychology, 15*(3), 340-363. <https://doi.org/doi.org/10.1521/jscp.1996.15.3.340>

Stice, E., Ziemba, C., Margolis, J., & Flick, P. (1996). The dual pathway model differentiates bulimics, subclinical bulimics, and controls: Testing the continuity hypothesis. *Behavior therapy, 27*(4), 531-549. <https://doi.org/doi.org/10.1016/S0005-7894(96)80042-6>

Tylka, T. L., & Wood-Barcalow, N. L. (2015). The Body Appreciation Scale-2: item refinement and psychometric evaluation. *Body Image, 12*, 53-67. <https://doi.org/doi.org/10.1016/j.bodyim.2014.09.006>
