# Supplementary material for: Assessing associations amongst body dissatisfaction, eating disorder symptoms and sociocultural influences in adolescents from rural Nicaragua
Source: BMC Psychol. 2025 Dec 6;14:32. doi: 10.1186/s40359-025-03728-3 (PMC12797590; doi:10.1186/s40359-025-03728-3)
Supplement: Supplementary file 2 — Supplementary Material 2 [file 40359_2025_3728_MOESM2_ESM.docx]

# **Supplementary Material S2 – ESEM fit indices for SATAQ**

**Table S2.1**. Fit indices from ESEM for the SATAQ subscales

|  | **Items** | **χ2** | **df** | **RMSEA** | **CFI** | **TLI** | **SRMR** | **Subscales** |
| --- | --- | --- | --- | --- | --- | --- | --- | --- |
| SATAQ | 13 | 78.4 | 42 | 0.084 | 0.987 | 0.976 | 0.031 | General, athletic internalization, pressure |

*Note.* Exploratory Structural Equation Model (ESEM) analysis conducted in Mplus with whole sample (*N* = 122); SATAQ = Sociocultural Attitudes Towards Appearance Questionnaire-3; χ2= chi-square; df = degrees of freedom; RMSEA = Root Mean Square Error of Approximation; CFI = Comparative Fit Index; TLI = Tucker-Lewis Index; SRMR = Standardized Root Mean Residual.
