# Supplementary material for: Assessing associations amongst body dissatisfaction, eating disorder symptoms and sociocultural influences in adolescents from rural Nicaragua
Source: BMC Psychol. 2025 Dec 6;14:32. doi: 10.1186/s40359-025-03728-3 (PMC12797590; doi:10.1186/s40359-025-03728-3)
Supplement: Supplementary file 3 — Supplementary Material 3 [file 40359_2025_3728_MOESM3_ESM.docx]

# Supplementary Material S3 – Multiple regressions with SATAQ athletic internalization subscale

Table S3.1. Multiple regression with Body satisfaction (BESAA) as outcome, with SATAQ athletic internalization and PSPS as outcomes, separately for boys and girls with standardized β-coefficients.

|  | **Girls** | | | **Boys** | | |
| --- | --- | --- | --- | --- | --- | --- |
|  | **Model 1** | **Model 2** | **Model 3** | **Model 1** | **Model 2** | **Model 3** |
| **Constant** | 4.374^***^ | 4.576^***^ | 2.863^***^ | 4.309^***^ | 4.524^***^ | 3.056^***^ |
| **SATAQ athl. int** | -0.182^***^ | -0.131^**^ | -0.164^***^ | -0.181^**^ | -0.123 | -0.133^*^ |
| **PSPS** |  | -0.204^***^ | -0.117 |  | -0.228^**^ | -0.240^***^ |
| **Resid Std. Error** | 0.563 | 0.541 | 0.493 | 0.571 | 0.545 | 0.445 |
| **Adjusted R2** | 0.097 | 0.165 | 0.308 | 0.085 | 0.166 | 0.444 |

*Note.* * *p* < .05, ** *p* < .01, *** *p* < .001. BESAA = Body Esteem Scale, SATAQ = Sociocultural Attitudes Towards Appearance Questionnaire, athletic internalization subscale, PSPS = Perceived Sociocultural Pressures Scale.

Table S3.2. Multiple regression with disordered eating attitudes (EAT) as outcome, with SATAQ athletic internalization, PSPS and BAS as outcomes, separately for boys and girls with standardized β-coefficients.

|  | **Girls** | | | **Boys** | | |
| --- | --- | --- | --- | --- | --- | --- |
|  | **Model 1** | **Model 2** | **Model 3** | **Model 1** | **Model 2** | **Model 3** |
| **Constant** | 25.939^***^ | 19.334^***^ | 27.466^***^ | 21.866^***^ | 16.443^***^ | 19.788 |
| **SATAQ athl. int** | 2.360^**^ | 0.693 | 0.461 | 3.128^**^ | 1.659 | 1.569 |
| **PSPS** |  | 6.659^***^ | 6.296^***^ |  | 5.758^***^ | 5.589^***^ |
| **BESAA** |  |  | -1.777 |  |  | -0.739 |
| **Adjusted R2** | 0.063 | 0.398 | 0.401 | 0.070 | 0.238 | 0.220 |
| **Res Std. Error** | 8.977 | 7.192 | 7.177 | 10.693 | 9.683 | 9.795 |

*Note.* * indicates *p* < .05. ** indicates *p* < .01. BESAA = Body Esteem Scale, SATAQ = Sociocultural Attitudes Towards Appearance Questionnaire, athletic internalization subscale, PSPS = Perceived Sociocultural Pressures Scale, EAT = Eating Attitudes Scale.
